# Self-reported sensory processing and social participation across adult ADHD symptom groups

**DOI:** 10.3389/fpsyt.2026.1928930

**Published:** 2026-09-29

**Authors:** Turki Aljuhani, Nourah S. Alflayyeh, Rahaf S. Aloqail, Hissah A. Alodan, Waad Aljurayyad

**Affiliations:** 1 Department of Occupational Therapy, College of Applied Medical Sciences, King Saud bin Abdulaziz University for Health Sciences, Riyadh, Saudi Arabia; 2 King Abdullah International Medical Research Center, Riyadh, Saudi Arabia; 3 Department of Rehabilitation Services, King Abdulaziz Medical City, Ministry of National Guard Health Affairs, Riyadh, Saudi Arabia

**Keywords:** adult ADHD, attention-deficit/hyperactivity disorder, Saudi Arabia, sensory processing, sensory profile, social participation

## Abstract

**Introduction:**

Sensory processing differences are increasingly recognized in adults with attention-deficit/hyperactivity disorder (ADHD), but their relationship with social participation remains understudied. This study examined sensory processing and social participation across three adult ADHD symptom groups and evaluated the association between sensory processing and social participation.

**Methods:**

This cross-sectional online survey included 209 adults aged 21–65 years, categorized into self-reported ADHD diagnosis (*n* = 91), elevated ADHD symptoms without a reported diagnosis (*n* = 66), and low ADHD symptoms (*n* = 52) groups. Participants completed the Adult/Adolescent Sensory Profile, the Social Participation subscale of the Sensory Processing Measure–2 Adult, and Part A of the Adult ADHD Self-Report Scale. Adjusted linear models compared groups while controlling for anxiety/depression status. Hierarchical multiple regression examined the additional variance in social participation explained by the four sensory processing quadrants.

**Results:**

Significant group differences were observed across all four sensory processing quadrants and social participation score. Adults with low ADHD symptoms generally reported fewer sensory processing differences and social participation difficulties than the other two groups. The self-reported ADHD diagnosis and elevated ADHD symptom groups differed significantly only in the low registration quadrant. The sensory processing quadrants increased the model for an additional 26% of the variance in social participation beyond ADHD group and anxiety/depression status (Δ*R*^2^ = .260, *p* <.001), increasing the total variance to 49.7%. Higher low registration (β = .46, *p* <.001) and sensation avoiding (β = .18, *p* = .037) were associated with greater social participation difficulties, whereas higher sensation seeking was associated with fewer difficulties (β = −.22, *p* <.001).

**Conclusion:**

The findings suggest that the self-reported ADHD diagnosis group and the elevated ADHD symptoms group show similar patterns of sensory processing and social participation, and that sensory processing may be relevant to understanding social participation in this population.

## Introduction

Attention-deficit/hyperactivity disorder (ADHD) is a neurodevelopmental disorder characterized by persistent patterns of inattention and/or hyperactivity-impulsivity that interfere with functioning (1). Although ADHD is often identified in childhood, symptoms and related functional difficulties frequently persist into adulthood (2). Global estimates suggest that adult ADHD remains common, with prevalence varying depending on whether ADHD is defined as persistent ADHD from childhood at 2.58% or symptomatic ADHD in adulthood at 6.76% (3). These figures correspond to approximately 139.84 million and 366.33 million affected adults worldwide, respectively. In Saudi Arabia, a systematic review of 14 studies reported a pooled ADHD prevalence of 12.4% (95% CI: 5.4%–26.0%) across the included populations (4). Self-report screening studies among Saudi adults and university students have also reported elevated levels of ADHD symptoms, although estimates vary across samples and assessment methods (5–7).

Adults with ADHD frequently experience co-occurring psychiatric conditions, particularly anxiety and depressive disorders (8–10). Although the mechanisms underlying these associations remain incompletely understood, evidence suggests contributions from overlapping genetic, neurobiological, and psychosocial pathways (11–13). These comorbidities may also complicate differential diagnosis because ADHD symptoms can overlap with those of mood and anxiety disorders (14).

Beyond psychiatric comorbidity, ADHD can affect multiple areas of daily functioning in adulthood, including difficulties in education, employment, emotional well-being, and quality of life (15–18). Previous studies have reported poorer educational and occupational outcomes, including reduced productivity, increased absenteeism, and lower employment rates (19, 20). Social participation, broadly defined as engagement in social activities and interactions across family, peer, community, and work contexts, represents another important domain of adult functioning (21). Specifically, participants reporting a prior ADHD diagnosis may experience difficulties in interpersonal relationships, perceived social support, and communication that can interfere with participation in these contexts (15–18). Pragmatic communication, defined as the context-appropriate use of language and conversational behavior, has received increasing attention in the ADHD literature (22). A systematic review and meta-analysis found poorer pragmatic language abilities among individuals with ADHD than among comparison participants without ADHD (23). Adult ADHD research has similarly identified difficulties regulating spoken interactions, including excessive talking, interrupting others, and speaking before thinking (24). Because social participation often occurs in complex environments involving simultaneous social and sensory demands, Dunn’s framework can provide a theoretical basis for considering whether differences in sensory thresholds and self-regulation strategies may influence how adults respond to and engage in these contexts (25–27).

Studies involving adults with ADHD have reported differences across all four sensory processing quadrants of low registration, sensation seeking, sensory sensitivity, and sensation avoiding, compared with adults without ADHD (28–31). According to Dunn’s model of sensory processing (27, 32), these patterns are determined by the interaction of the neurological threshold (low/high) and behavioral response strategy (active/passive). Sensory sensitivity and sensation avoidance both reflect a low neurological threshold, resulting in the nervous system being highly responsive to sensory stimuli. Individuals with sensory sensitivity respond passively and may experience discomfort when exposed to sensory stimuli, but those with sensory avoidance engage in active behavioral strategies (e.g., leaving the crowd area) to reduce or avoid sensory input. In contrast, individuals with low registration and sensation seeking reflect a high neurological threshold in which the nervous system, requires intense sensory stimuli, but they differ in self-regulation strategy: low-registration relates to passive self-regulation, resulting in reduced awareness of sensory inputs and delayed response to environmental stimuli, whereas sensation seeking involves actively pursuing additional or more intense sensory experiences (32).

These sensory processing patterns may shape how individuals with ADHD notice, tolerate, and respond to environmental demands and may influence attention, emotional regulation, sleep, work performance, and participation in daily activities (33, 34). Despite growing recognition, sensory processing remains underrepresented in adult ADHD research relative to core symptom domains, and little is known about how specific sensory patterns relate to social participation. Existing studies have also largely compared adults with and without ADHD, providing limited insight into adults with elevated ADHD symptoms who do not report a diagnosis (30, 35).

Accordingly, the aim of this study was twofold: first, to compare sensory processing and social participation among adults with a self-reported ADHD diagnosis, adults with elevated ADHD symptoms but no reported diagnosis, and adults with low ADHD symptoms; and second, to examine associations between sensory processing patterns and social participation. We hypothesized that sensory processing patterns would be associated with social participation difficulties.

## Materials and methods

### Study design and participants

This descriptive cross-sectional study used convenience sampling to recruit adults aged 21–65 years between September 2024 and August 2025. Participants were excluded if they reported autism spectrum disorder, hearing impairment, or a speech or language disorder because these conditions were considered to potentially affect sensory processing and social participation independently, thereby complicating interpretation of differences associated with ADHD symptom status (36–38). Other self-reported psychiatric and learning-related conditions were not exclusionary to preserve the clinical representativeness of the sample. The final analytical sample included 209 participants across the three study groups.

### Data collection procedure

Participants were recruited from King Abdulaziz Medical City in Riyadh and through the Saudi ADHD Society via electronic invitations and digital outreach. Questionnaires were distributed through an online survey platform. Duplicate submissions were monitored and removed manually during the data screening and cleaning phase prior to statistical analysis. Data collection began after ethical approval was obtained from the Institutional Review Board of King Abdullah International Medical Research Center (IRB Approval No. IRB/0994/24; Study No. SP24R/051/03) and the Saudi ADHD Society (RP24007/HO-01-R119). Informed consent was obtained from all participants before participation. Confidentiality, privacy, and adherence to ethical research standards were maintained throughout the study.

### Outcome measures

Participants provided demographic and clinical information, including age, gender, education level, comorbid conditions, and ADHD diagnostic history. Participants who self-reported a prior ADHD diagnosis were also asked to report the age of diagnosis and treatment history. Diagnostic history was assessed using the item: “Have you been formally diagnosed with Attention-Deficit/Hyperactivity Disorder (ADHD)?” (Yes/No), followed by, if yes: “At what age were you diagnosed with ADHD?”.

Three outcome measures were used: the Adult/Adolescent Sensory Profile (AASP), the Social Participation subscale of the Sensory Processing Measure-2 Adult Self-Report (SPM-2 Adult), and the Adult ADHD Self-Report Scale (ASRS). All outcome measures were presented in a dual-language format, with the validated English and Arabic text displayed simultaneously for each item.

#### Arabic adult/adolescent sensory profile

The AASP was used to assess sensory processing patterns in daily life. The AASP is a 60-item self-report measure that examines responses to everyday sensory experiences across six sensory categories: taste/smell, movement, visual, touch, activity level, and auditory processing (39). The measure is based on Dunn’s model of sensory processing and yields four sensory processing patterns: low registration, sensation seeking, sensory sensitivity, and sensation avoiding quadrant (27, 32). Higher scores indicate greater endorsement of the corresponding sensory processing pattern. The validated Arabic version of the AASP demonstrated acceptable internal consistency, with an overall Cronbach’s = .78, and cross-language item agreement ranging from 77% to 98% compared with the original English version, supporting its linguistic equivalence and use among Arabic-speaking individuals (40). In the present sample, internal consistency across the four sensory processing quadrants ranged from α = .66 to.88 (**Supplementary Table S1**).

#### Social participation subscale

Social participation was assessed using the Social Participation subscale of the SPM-2 Adult (41, 42), a self-report measure intended for adults aged 21–87 years. The subscale assesses the self-reported frequency of behaviors reflecting participation in social activities and the ability to interact effectively with others in everyday settings, including group involvement and getting along with other people. Items are rated on a 4-point Likert scale ranging from never to always. Items 1, 2, 4, 5, and 10 indicate better social participation and were reverse-coded before summing all 10 items, with higher total scores indicating greater social participation challenges. In the original English SPM-2 Adult, internal consistency across domains ranged from α = .79 to.95, with α = .80 reported for Social Participation (42). A recent psychometric evaluation of the Arabic SPM-2 Adult reported low internal consistency for the Social Participation subscale (α = .18), despite stronger reliability for the overall scale (43). In the present sample, the 10-item Social Participation subscale demonstrated acceptable internal consistency (Cronbach’s α = .77; **Supplementary Table S1**).

#### Adult ADHD self-report scale

Adult ADHD symptoms were assessed using the ASRS v1.1 Symptom Checklist, which aligns with the *Diagnostic and Statistical Manual of Mental Disorders*, Fourth Edition, Text Revision (DSM-IV-TR) criteria (44). While the full instrument contains 18 items, this study utilized the official 6-item ASRS v1.1 Screener (Part A), which consists of the six questions most predictive of adult ADHD. Items are rated on a 5-point Likert scale ranging from “never” to “very often”. Participants completed either the official English version or the cross-translated Arabic version (45). Previous psychometric evaluation demonstrated strong internal consistency (Cronbach’s α = 0.88) and agreement with clinician-administered ratings (ICC = 0.84) (46). Recent Arabic-language psychometric evaluations supported a single-factor structure and acceptable internal consistency for both the DSM-5-based ASRS-5-AR (Cronbach’s α = 0.788) and the ASRS-v1.1 six-item screener (Cronbach’s α = 0.68) (47, 48). In the present sample, the six-item ASRS Part A demonstrated acceptable internal consistency (Cronbach’s α = .75; **Supplementary Table S1**). Following standardized scoring guidelines, endorsement of four or more items at their designated frequency thresholds was considered indicative of elevated ADHD symptoms rather than a clinical diagnosis.

### ADHD symptom group classification

After survey completion, participants were categorized into three groups. Participants who reported a previous clinical ADHD diagnosis were assigned to the self-reported ADHD diagnosis group, regardless of their current ASRS Part A score. Participants without a self-reported ADHD diagnosis were classified into the elevated ADHD symptoms group if they scored 4 or higher on ASRS Part A, and into the low ADHD symptoms group if they scored below 4. Because the ASRS is a screening instrument, participants meeting the cutoff were not considered to have a confirmed ADHD diagnosis.

### Statistical analysis

All analyses were conducted in R version 4.2.2 using a two-sided α of.05. Data were screened for missingness, coding errors, and out-of-range values. Categorical variables were summarized using frequencies and percentages and continuous variables using means and standard deviations or medians and interquartile ranges, as appropriate. Because continuous demographic and clinical variables showed departures from normality based on graphical inspection and Shapiro–Wilk tests, group differences were examined using Kruskal–Wallis tests; categorical variables were compared using Pearson’s chi-square tests.

Adjusted group differences in the four AASP sensory quadrants and social participation were examined using separate linear models with study group and anxiety/depression status as categorical predictors. Anxiety and depression were combined into a dichotomous indicator because of substantial overlap and sparse group-specific cells; the cross-classification of anxiety and depression by study group is presented in (**Supplementary Table S2**). To evaluate whether this combined specification affected our results, we conducted a sensitivity analysis entering anxiety and depression as separate covariates in these group-comparison models. Benjamini–Hochberg false discovery rate correction was applied across the AASP outcomes; social participation was treated separately.

Hierarchical multiple linear regression was used to examine associations between sensory processing and social participation. Model 1 included study group and anxiety/depression status. Model 2 added all four sensory processing quadrants simultaneously. As a sensitivity analysis, this hierarchical regression was also conducted with anxiety and depression entered as separate covariates rather than the combined indicator. Model assumptions were assessed using post-estimation diagnostics. Linearity and homoscedasticity were assessed using residual-versus-fitted plots and the Breusch–Pagan test. Residual normality was assessed using Q–Q plots and the Shapiro–Wilk test, and residual independence using the Durbin–Watson test. Multicollinearity was evaluated using variance inflation factors and influential observations using Cook’s distance (>1 considered potentially influential).

Effect sizes were interpreted, using conventional benchmarks for Partial η^2^, Cohen’s *d*, and Cohen’s *f*^2^, as heuristic guidelines rather than thresholds for clinical significance (49). *Post-hoc* sensitivity analyses based on the achieved sample of 209 participants, α = .05, and 80% power indicated detectable effects of approximately Cohen’s *f* = 0.22 (partial η^2^ ≈ 0.04) for adjusted group comparisons and Cohen’s *f*^2^ = 0.06 for the hierarchical regression, using G*Power version 3.1.9.7.

## Results

### Participant characteristics

The final analytic sample included 209 participants: 91 in the self-reported ADHD diagnosis group, 66 in the elevated ADHD symptoms group, and 52 in the low ADHD symptoms group. Participant characteristics are summarized in **Table 1**, and the participant flow is presented in **Figure 1**.

**Table 1:** Participant characteristics.

Characteristic	Self-reported ADHD diagnosis N = 91	Elevated ADHD symptoms N = 66	Low ADHD symptoms N = 52	Overall N=209^1^	*p* ^2^
Age	27.0 (23.0, 34.0); range: 21.0–46.0	25.0 (22.0, 34.0); range: 21.0–61.0	28.5 (21.5, 36.5); range: 21.0–64.0	27.0 (22.0, 34.0); range: 21.0–64.0	.709
Gender					.492
Male	27 (29.7%)	24 (36.4%)	14 (26.9%)	65 (31.1%)	
Female	64 (70.3%)	42 (63.6%)	38 (73.1%)	144 (68.9%)	
National background					.794
Saudi	80 (87.9%)	57 (86.4%)	47 (90.4%)	184 (88.0%)	
Non-Saudi/other	11 (12.1%)	9 (13.6%)	5 (9.6%)	25 (12.0%)	
Education level					.737
No formal education	0 (0.0%)	0 (0.0%)	1 (1.9%)	1 (0.5%)	
High school/diploma	21 (23.1%)	15 (22.7%)	15 (28.8%)	51 (24.4%)	
Bachelor’s degree	58 (63.7%)	43 (65.2%)	31 (59.6%)	132 (63.2%)	
Graduate or professional degree	12 (13.2%)	8 (12.1%)	5 (9.6%)	25 (12.0%)	
Smoking status					.346
Non-smoker	66 (72.5%)	52 (78.8%)	43 (82.7%)	161 (77.0%)	
Current smoker	25 (27.5%)	14 (21.2%)	9 (17.3%)	48 (23.0%)	
Smoking frequency among current smokers					
Less than once a week	0 (0.0%)	3 (21.4%)	1 (11.1%)	4 (8.3%)	
1–2 times a week	0 (0.0%)	1 (7.1%)	1 (11.1%)	2 (4.2%)	
3–6 times a week	1 (4.0%)	0 (0.0%)	1 (11.1%)	2 (4.2%)	
Daily	24 (96.0%)	10 (71.4%)	6 (66.7%)	40 (83.3%)	
Anxiety	57 (62.6%)	20 (30.3%)	4 (7.7%)	81 (38.8%)	<.001**
Depression	46 (50.5%)	10 (15.2%)	4 (7.7%)	60 (28.7%)	<.001**
Other diagnoses	9 (9.9%)	2 (3.0%)	0 (0.0%)	11 (5.3%)	.018*
Family member diagnosed ADHD	31 (34.1%)	10 (15.2%)	6 (11.5%)	47 (22.5%)	.002*
Family member suspected ADHD	77 (84.6%)	47 (71.2%)	17 (32.7%)	141 (67.5%)	<.001**

Median (Q1, Q3); range: Min–Max; n (%).

2 Kruskal-Wallis rank sum test; Pearson’s Chi-squared test with simulated p-value (based on 2000 replicates). * p < 0.05; ** p < 0.001.

**Figure 1:**
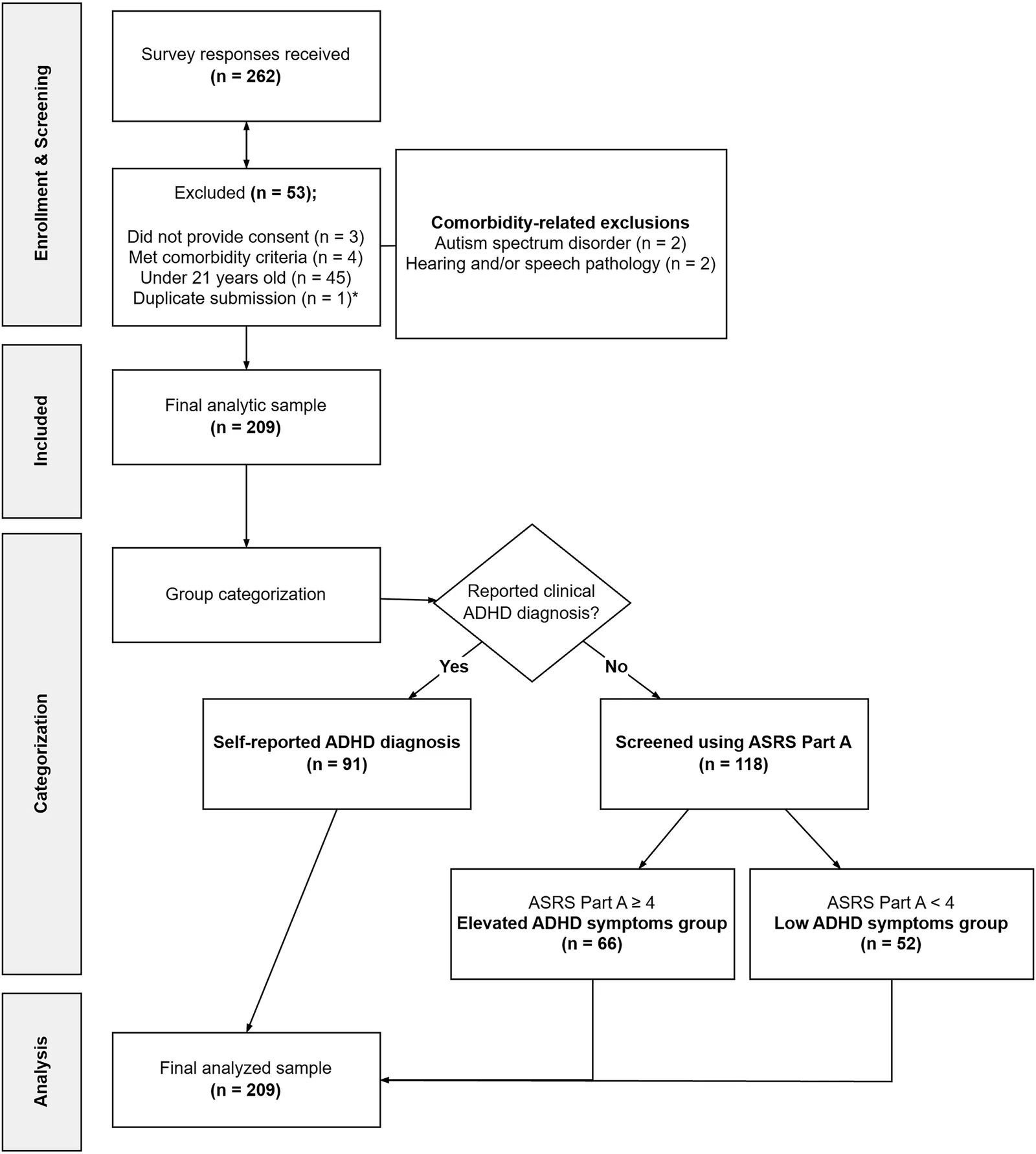
Participant flow diagram. Participants were screened for eligibility and categorized based on self-reported ADHD diagnosis and ASRS Part A scores. Exclusion categories were mutually exclusive. *Duplicate submissions were identified manually.

Age was similar between the three groups (p = .7). The median age of the overall sample was 27 years (range: 21- 64). Most participants across all groups were female (68.9%), Saudi (88.0%), and held a bachelor’s degree (63.2%). Smoking frequency differed significantly across groups (*p* = <.001), with daily frequency smoking reported most often in the self-reported ADHD diagnosis group (24 out of 25 participants, 96.0%).

Clinical characteristics varied significantly across the groups. Reported anxiety and depression diagnoses were most frequent in the self-reported ADHD diagnosis group (62.6% and 50.5%, respectively), followed by the elevated ADHD symptoms group (30.3% and 15.2%) and the low ADHD symptoms group (7.7% for both). Other reported comorbid diagnoses also differed significantly across groups (*p* = .018), reported primarily in the self-reported ADHD diagnosis group (9.9%). Family history of both diagnosed and suspected ADHD differed significantly across groups (p = 0.04, p ≤.001), with the highest proportions reported by participants in the self-reported ADHD diagnosis group (34.1% and 84.6%, respectively).

Among participants with a self-reported ADHD diagnosis, the median reported age of formal diagnosis was 25 years (range: 5 – 45). A total of 79 participants (86.8%) reported a history of ADHD treatment. Stimulant medication use was the most common treatment approach (70.0%), followed by cognitive behavioral therapy, behavioral therapy, and non-stimulant medication use (**Supplementary Table S3**).

Screening status cross-tabulation showed that most participants in the self-reported ADHD diagnosis group scored at or above the ASRS Part A screening threshold of 4 (88/91, 96.7%), whereas three participants (3.3%) scored below the threshold (**Supplementary Table S4**). Because group classification was based on self-reported prior ADHD diagnosis rather than concurrent ASRS screening status, these participants remained in the self-reported diagnosis group. For participants who indicated they did not have a prior clinical diagnosis, a follow-up question assessed whether they self-suspected having ADHD. Among those who suspected that they had ADHD, 53 of 66 participants (85.5%) scored at or above the screening threshold. In contrast, 13 of 52 participants (23.2%) who did not suspect that they had ADHD scored at or above the threshold.

### Group differences in sensory processing and social participation

Residual-versus-fitted plots indicated no substantial violations of homoscedasticity. Multicollinearity was low, with all variance inflation factors below 1.5. Cook’s distances indicated no unduly influential observations, with a maximum value of 0.06. Residuals were normally distributed across models.

#### Sensory processing quadrants

Models adjusted for anxiety/depression status showed significant group differences across all four sensory processing quadrants after Benjamini–Hochberg correction (all adjusted *p* <.007; **Table 2**; **Figure 2**). Effect sizes ranged from partial η^2^ = .050 to.404, with the largest group effect observed for low registration, F(2, 205) = 41.79, *p* <.001, partial η^2^ = .404.

**Table 2:** Adjusted group differences in sensory processing and social participation.

Outcome	Self-reported ADHD diagnosis	Elevated ADHD symptoms	Low ADHD symptoms	F (df1, df2)	p	Partial η^2^	*p_adj_*
Low registration	32.47 (29.79, 35.14)	48.43 (46.48, 50.37)	43.79 (41.56, 46.01)	41.79 (2, 205)	<.001**	0.404	<.001**
Sensation seeking	45.14 (42.82, 47.45)	50.06 (48.38, 51.74)	47.95 (46.02, 49.88)	5.12 (2, 205)	.007*	0.050	.007*
Sensory sensitivity	42.53 (40.05, 45.01)	51.23 (49.43, 53.03)	53.35 (51.29, 55.42)	25.12 (2, 205)	<.001**	0.288	<.001**
Sensation avoiding	43.55 (40.94, 46.17)	52.06 (50.16, 53.96)	51.66 (49.48, 53.84)	15.11 (2, 205)	<.001**	0.225	<.001**
Social participation	22.83 (20.99, 24.67)	29.88 (28.54, 31.22)	28.22 (26.69, 29.75)	17.75 (2, 205)	<.001	0.238	—

Values are adjusted means with 95% confidence intervals. F tests and partial η^2^ represent the adjusted omnibus study-group effect. BH-adjusted p values were calculated across the four AASP quadrant outcomes using the Benjamini–Hochberg false discovery rate procedure; Social Participation was evaluated separately and not included in this correction. * p < 0.05; ** p < 0.001.

**Figure 2:**
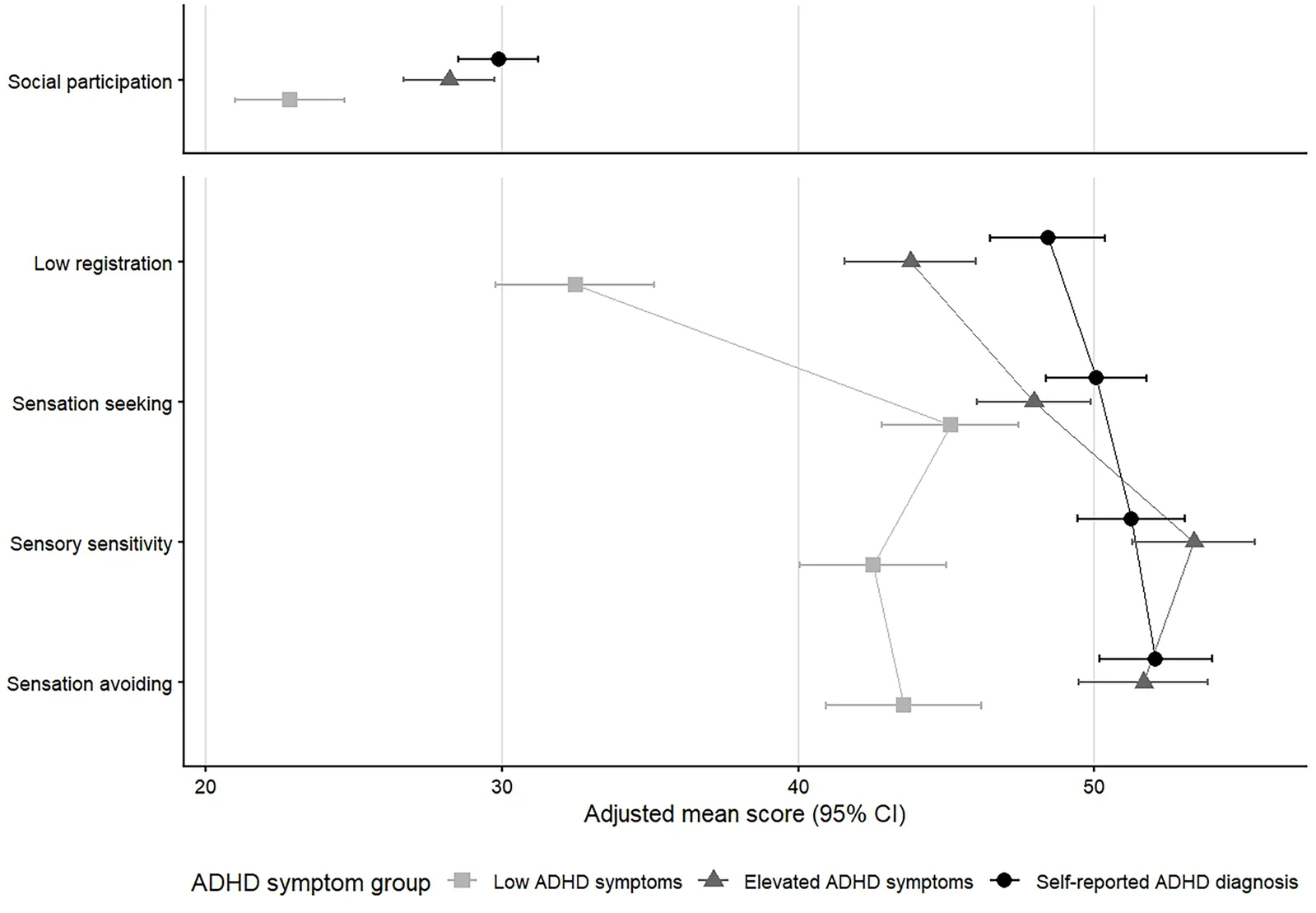
Adjusted group mean scores with 95 percent confidence intervals for social participation difficulties and sensory processing quadrants across the three ADHD study groups. Values are derived from separate linear regression models adjusted for anxiety and/or depression diagnosis. Social participation difficulties are scored on a scale ranging from 10 to 50, where higher scores indicate greater difficulty. Adolescent/Adult Sensory Profile quadrant scores range from 15 to 75, where higher scores reflect a greater frequency of the sensory processing style.

*Post hoc* pairwise comparisons showed different patterns across the quadrants (**Table 3**). For low registration, scores differed significantly among all three groups, with the self-reported ADHD diagnosis group scoring highest, followed by the elevated ADHD symptoms group and the low ADHD symptoms group. For sensory sensitivity and sensation avoiding, the low ADHD symptoms group had significantly lower scores than both the self-reported ADHD diagnosis and elevated ADHD symptoms groups, with no significant difference between the latter two groups (both p >.05). For sensation seeking, the low ADHD symptoms group had only significantly lower scores than the self-reported ADHD diagnosis group. Effect sizes for the significant pairwise comparisons ranged from |Cohen’s *d*| =0.52 to 1.79, indicating approximately medium to large effects.

**Table 3:** Adjusted pairwise comparisons of sensory processing quadrants and social participation across study groups.

Outcome	Contrast	Mean difference (95% CI)	Cohen’s d (95% CI)	t	*p_adj_*
Low registration	Low ADHD symptoms - (Self-reported ADHD)	-15.96 (-20.15, -11.76)	-1.79 (-2.22, -1.36)	-8.98	<.001**
	Low ADHD symptoms - Elevated ADHD symptoms	-11.32 (-15.29, -7.35)	-1.27 (-1.66, -0.88)	-6.73	<.001**
	(Self-reported ADHD) - Elevated ADHD symptoms	4.64 (0.97, 8.30)	0.52 (0.17, 0.87)	2.99	.009*
Sensation seeking	Low ADHD symptoms - (Self-reported ADHD)	-4.92 (-8.55, -1.29)	-0.64 (-1.03, -0.24)	-3.20	.005*
	Low ADHD symptoms - Elevated ADHD symptoms	-2.82 (-6.25, 0.62)	-0.36 (-0.74, 0.01)	-1.93	.132
	(Self-reported ADHD) - Elevated ADHD symptoms	2.11 (-1.07, 5.28)	0.27 (-0.07, 0.62)	1.57	.262
Sensory sensitivity	Low ADHD symptoms - (Self-reported ADHD)	-8.70 (-12.59, -4.82)	-1.05 (-1.46, -0.65)	-5.29	<.001*
	Low ADHD symptoms - Elevated ADHD symptoms	-10.82 (-14.50, -7.14)	-1.31 (-1.70, -0.92)	-6.95	<.001*
	(Self-reported ADHD) - Elevated ADHD symptoms	-2.12 (-5.51, 1.28)	-0.26 (-0.60, 0.09)	-1.47	.307
Sensation avoiding	Low ADHD symptoms - (Self-reported ADHD)	-8.51 (-12.61, -4.41)	-0.97 (-1.38, -0.57)	-4.90	<.001*
	Low ADHD symptoms - Elevated ADHD symptoms	-8.11 (-11.99, -4.22)	-0.93 (-1.31, -0.55)	-4.93	<.001*
	(Self-reported ADHD) - Elevated ADHD symptoms	0.40 (-3.18, 3.99)	0.05 (-0.30, 0.39)	0.27	.962
Social participation	Low ADHD symptoms - (Self-reported ADHD)	-7.05 (-9.93, -4.16)	-1.15 (-1.55, -0.74)	-5.76	<.001*
	Low ADHD symptoms - Elevated ADHD symptoms	-5.39 (-8.12, -2.66)	-0.88 (-1.26, -0.50)	-4.66	<.001*
	(Self-reported ADHD) - Elevated ADHD symptoms	1.66 (-0.86, 4.18)	0.27 (-0.07, 0.61)	1.55	.269

Mean differences are based on adjusted estimated marginal means from models controlling for anxiety/depression status. P values were adjusted using Tukey’s method within each outcome. Cohen’s d was standardized using the residual standard deviation from the corresponding adjusted model. * p < 0.05; ** p < 0.001.

#### Social participation difficulties

Significant group differences were observed in social participation difficulties, *F* (2, 205) = 17.75, *p* <.001, partial η^2^ = .238. (**Table 2**). Tukey-adjusted pairwise comparisons showed that the low ADHD symptoms group had lower scores than the self-reported ADHD diagnosis group (mean difference = -7.05, 95% CI [-9.93, -4.16], p <.001) and the elevated ADHD symptoms group (mean difference = −5.39, 95% CI [−8.12, −2.68], p <.001), indicating fewer social participation difficulties. The self-reported ADHD diagnosis and elevated ADHD symptoms groups did not differ significantly (mean difference = 1.66, 95% CI [−0.86, 4.18], p = .269). Effect sizes for the significant comparisons were large (|Cohen’s d| = .88–1.15; **Table 3**).

### Associations between sensory processing and social participation

A hierarchical multiple linear regression examined associations between sensory processing quadrants and social participation difficulties accounting for ADHD group and anxiety/depression status (**Table 4**). Model 1 explained 25.5% of the variance in social participation, *R*^2^ = .255, *F* (3, 205) = 23.35, *p* <.001. Relative to the low ADHD symptoms group, self-reported ADHD diagnosis (β = 1.0, *p* <.001) and elevated ADHD symptoms (β = 0.76, *p* <.001) were associated with greater social participation difficulties. Anxiety/depression status was also positively associated with social participation difficulties (β = 0.35, *p* = .014).

**Table 4:** Hierarchical regression predicting social participation.

Predictor	Model 1: covariates only				Model 2: combined sensory model			
	B	SE	β	*p*	B	SE	β	*p*
Intercept	21.60	0.86	-0.83	<.001**	14.43	2.83	-0.19	<.001**
Self-reported ADHD diagnosis	7.05	1.22	1.00	<.001**	1.97	1.18	0.28	.097
Elevated ADHD symptoms	5.39	1.16	0.76	<.001**	1.13	1.08	0.16	.298
Anxiety/depression diagnosis	2.46	0.99	0.35	.014*	0.24	0.85	0.03	.774
q1: Low registration	—	—	—	—	0.28	0.05	0.46	<.001**
q2: Sensation seeking	—	—	—	—	-0.19	0.05	-0.22	<.001**
q3: Sensory sensitivity	—	—	—	—	0.06	0.06	0.08	.355
q4: Sensation avoiding	—	—	—	—	0.12	0.06	0.18	.037*
Model fit summary								
R^2^	.255				.514			
Adjusted R^2^	.244				.497			
F statistic	F (3, 205) = 23.35, p <.001**				F (7, 201) = 30.40, p <.001**			
ΔR^2^	—				.260			
ΔF	—				F (4, 201) = 26.85, p <.001**			

N = 209. Outcome, social participation score; B, unstandardized regression coefficient; SE, standard error; β, standardized regression coefficient; R^2^, proportion of variance explained by the model; adjusted R^2^, R^2^ adjusted for the number of predictors; ΔR^2^, change in explained variance between models; ΔF, F test of the change in R^2^ between models. Study group was dummy-coded with Low ADHD Symptoms as the reference category, and anxiety/depression status was coded with No as the reference category. Variance inflation factors (VIFs) for all predictors in Model 2 were below standard thresholds (maximum VIF = 3.43). * p < 0.05; ** p < 0.001.

Adding the four sensory processing quadrants in Model 2 explained an additional 26.0% of the variance in social participation, Δ*R*^2^ = .260, Δ*F* (4, 201) = 26.85, *p* <.001. The final model explained 51.4% of the variance, *R*^2^ = .514, *F* (7, 201) = 30.40, *p* <.001, *f*^2^ = 1.06. Higher low registration (β = 0.46, *p* <.001) and sensation avoiding scores (β = 0.18, *p* = .037) were uniquely associated with greater social participation difficulties, whereas higher sensation seeking scores were associated with fewer difficulties (β = −0.22, *p* <.001). Sensory sensitivity was not independently associated with social participation after accounting for the other sensory quadrants and covariates (β = 0.08, *p* = .355). The coefficients for self-reported ADHD diagnosis, elevated ADHD symptoms, and anxiety/depression status were attenuated and were no longer statistically significant after the sensory quadrants were added.

Post-estimation diagnostics indicated no major model violations. Multicollinearity was acceptable, with a maximum variance inflation factor of 3.43. Diagnostic tests did not indicate heteroscedasticity (*p* = .543), residual dependence (*p* = .954), or a substantial departure from residual normality (*p* = .055).

Results were consistent when anxiety and depression were entered as separate covariates rather than the combined indicator: omnibus study-group effects for the group-comparison models were nearly unchanged across all outcomes (**Supplementary Table S5**), and AASP quadrant coefficients in the hierarchical regression remained stable (**Supplementary Table S6**).

## Discussion

This study examined sensory processing and social participation among adults with a self-reported ADHD diagnosis, elevated ADHD symptoms, and low ADHD symptoms. By accounting for anxiety/depression status, the findings provide insight into how sensory processing patterns and ADHD symptom status are associated with social participation difficulties.

A key finding was that sensory processing differences were not confined to participants with a self-reported ADHD diagnosis. Both the self-reported ADHD diagnosis and elevated ADHD symptoms groups reported significantly higher scores than the low ADHD symptoms group on low registration, sensory sensitivity, and sensation avoiding. For sensation seeking, only the self-reported ADHD diagnosis group differed significantly from the low ADHD symptoms group. These findings are consistent with previous studies documenting sensory processing differences between adults with and without ADHD (29, 31). The self-reported ADHD diagnosis and elevated ADHD symptoms groups did not differ significantly from one another on sensation seeking, sensory sensitivity, or sensation avoiding, whereas low registration was significantly higher in the self-reported ADHD diagnosis group. Together, this pattern suggests that sensory processing differences may accompany elevated ADHD symptoms regardless of reported diagnostic status (50).

The self-reported sensory patterns observed in this adult sample broadly align with findings from younger ADHD samples (51–53). Previous research has identified low registration, activity level, and auditory processing as areas that differentiate adolescents with ADHD from those without ADHD (53). In the present study, low registration was the only sensory outcome that distinguished the self-reported ADHD diagnosis group from the elevated ADHD symptoms group, and it also showed the largest overall group effect (partial η^2^ = 0.404), suggesting that low registration may be a distinguishing characteristic of adults with a self-reported ADHD diagnosis relative to those with elevated symptoms.

A similar group pattern emerged for social participation. Participants in both the self-reported ADHD diagnosis and elevated ADHD symptoms groups reported greater social participation difficulties than those in the low ADHD symptoms group, after accounting for anxiety/depression status. This finding aligns with previous research documenting difficulties in social relationships, communication, and participation in everyday contexts among participants with clinical or reported ADHD diagnoses (54–56). Social participation scores did not differ significantly between the self-reported ADHD diagnosis and elevated ADHD symptoms groups. This parallel pattern suggests that social participation difficulties may also be present among adults with elevated ADHD symptoms who do not report a formal diagnosis, and may be more closely related to the elevated symptoms and associated functional characteristics than to reported diagnostic status alone (57). Importantly, lower or more selective participation may not necessarily reflect impairment. Evidence from the broader adult sensory-processing literature suggests that individuals may intentionally limit exposure to overwhelming environments or select more predictable settings as a strategy for regulating sensory demands (58–60).

This distinction is particularly relevant when interpreting the associations between sensory processing and social participation. A recent systematic review and meta-analysis confirmed a greater atypical sensory processing across all four quadrants among individuals with ADHD compared with control populations, supporting sensory processing as a clinically relevant dimension of ADHD (30). Extending this evidence, adding the four sensory-quadrant scores to the regression model increased R^2^ by 26.0 percentage points (from 23.7% to 49.7%) in social participation, beyond ADHD group and anxiety/depression status. Of the four quadrants, low registration, sensation seeking, and sensation avoiding each remained significant unique predictors in the full model. After the sensory quadrants were added, the coefficients for ADHD group and anxiety/depression status were substantially attenuated and no longer statistically significant. If replicated, these results suggest that the associations of ADHD group and anxiety/depression status with social participation are not independent of the sensory processing variables in the model. We note, however, that the cross-sectional nature of this analysis precludes any definitive conclusions regarding directionality or causality. Although the sensory quadrants made a substantial statistical contribution at the sample level, the study did not evaluate clinically meaningful thresholds, functional impairment criteria, or minimally important differences for the AASP or SPM-2 Social Participation scores. Therefore, higher sensory quadrant scores and their associations with social participation should not be interpreted as evidence of clinically significant impairment at the individual level. Further research using clinically anchored functional measures is needed to determine the practical and individual-level significance of these associations.

Overall, the pattern observed in the present study closely parallels broader adult occupational-engagement research, in which higher low registration, sensory sensitivity, and sensation avoiding were associated with lower engagement, whereas higher sensation seeking was associated with greater engagement (61). Within Dunn’s framework, low registration and sensation seeking both reflect a high neurological threshold, differing in behavioral response: low registration involves a passive response, such that sensory input may be noticed less readily or responded to more slowly, whereas sensation seeking involves actively pursuing additional sensory input. Sensory sensitivity and sensation avoiding, by contrast, both reflect a low neurological threshold: sensory sensitivity involves a passive response to readily noticed sensory input, while sensation avoiding reflects active efforts to limit exposure to overwhelming or unpredictable environments. Together, these patterns suggest that social participation may be shaped by how individuals notice, regulate, and approach sensory experiences across this threshold-and-response structure. These mechanisms, however, were not directly examined in this study.

The demographic characteristics of the sample provide important context for interpreting the findings. Among participants with a self-reported ADHD diagnosis, the median reported age at diagnosis was 27 years, and females comprised 68.9% of the overall sample. Although ADHD was historically recognized more often in males and during childhood, increasing numbers of adults, particularly women, are receiving diagnoses later in life (2, 62, 63). The predominance of women in the present sample may reflect this broader diagnostic trend and should also be considered when evaluating the generalizability of the findings. Among participants without a reported ADHD diagnosis who suspected they had ADHD, 85.5% met the ASRS Part A screening threshold. Although this finding does not indicate a clinical diagnosis, it may highlight the need for clearer pathways to comprehensive adult ADHD evaluation and suggests a possible gap in adult ADHD identification in this population (64, 65).

Our cross-sectional data also revealed high rates of co-occurring mental health diagnoses within the sample. Reported anxiety and depression were most frequent in the self-reported ADHD diagnosis group, followed by the elevated ADHD symptoms group. This pattern is consistent with regional studies and broader systematic-review evidence on psychiatric comorbidity in adult ADHD (5, 6, 8). A small number of participants reported other psychiatric or learning-related conditions, including bipolar disorder, borderline personality disorder, learning disabilities, and schizophrenia, as detailed in **Table 1**. Symptoms associated with anxiety, depression, and other psychiatric conditions may overlap with ADHD-related inattention, restlessness, and executive difficulties, potentially contributing to elevated self-reported ASRS scores and complicating interpretation of screening results (14, 66).

To our knowledge, this is the first study to examine sensory processing and social participation across different levels of reported ADHD diagnostic and symptom status. The relatively large sample and use of widely used, psychometrically supported measures, together with evaluation of internal consistency in the present sample, strengthen the methodological rigor of the study. Adjusting for co-occurring anxiety/depression allowed examination of group differences and associations beyond this factor. The study also contributes evidence from an understudied Saudi and Arabic-speaking adult population.

This study has some limitations. The cross-sectional design provides only a snapshot of participants’ current experiences and precludes conclusions about directionality or causality. ADHD diagnosis and all primary study variables were self-reported, and diagnostic status was not independently verified, introducing potential recall and response bias (67). Because all measures were completed within the same survey context, observed associations may also partly reflect shared response tendencies or negative affectivity rather than exclusively reflecting associations between sensory processing and social participation (68). Moreover, because all measures were presented in a simultaneous dual-language format (Arabic and English), our reliability estimates may not be directly comparable to those from single-language validation studies (43). Additionally, treatment history was available only for participants reporting an ADHD diagnosis and therefore could not be evaluated as a potential confounder across all study groups. Other potentially relevant factors, including broader medical history, socioeconomic status, functional impairment, and neurodevelopmental characteristics such as autism traits, were not assessed. Although anxiety/depression status was included as a covariate, residual confounding from these unmeasured factors remains possible. Finally, findings from the predominantly Saudi sample (88%) may reflect culturally specific patterns in social participation and mental health help-seeking (69). Convenience sampling, online administration, and recruitment via the Saudi ADHD Society may further limit generalizability and introduce selection bias.

Measurement-related limitations also warrant consideration. Use of the ASRS screening threshold to classify participants without a self-reported ADHD diagnosis may have introduced misclassification and obscured variation in continuous symptom severity (44). Sensory processing was assessed using the AASP, whereas social participation was measured using an SPM-2 Adult subscale situated within a sensory-processing framework. Because both measures are self-reported and conceptually related, regression associations may partly reflect shared conceptual content and common-method variance (70). Finally, clinically meaningful thresholds or minimally important differences were not evaluated; therefore, statistically significant findings should not be assumed to represent clinically meaningful individual-level differences.

Future research should use longitudinal and multimethod designs to clarify the direction of associations between sensory processing and social participation and reduce reliance on shared self-report methods. To better distinguish ADHD-related sensory and participation patterns from those associated with conditions presenting with overlapping symptoms, future studies should include clinically characterized comparison groups, such as adults with anxiety, depression, autism, or other major psychiatric conditions without ADHD. Such designs should also examine whether continuous ADHD symptom severity scores demonstrate graded associations with sensory processing and social participation across the full sample. Replication in larger and more culturally diverse samples is also needed. Finally, future studies should examine whether sensory processing patterns predict clinically meaningful changes in everyday participation and whether these patterns represent modifiable targets for individualized environmental or occupational interventions.

## Conclusion

Adults with a self-reported ADHD diagnosis and those with elevated ADHD symptoms showed greater sensory processing differences and social participation difficulties than adults with low ADHD symptoms. Self-reported sensory processing quadrants increased variance in social participation beyond ADHD group and anxiety/depression status, with low registration, sensation avoiding, and sensation seeking making unique contributions to the final model. Together, these findings show that adults with a self-reported ADHD diagnosis and those with elevated ADHD symptoms display similar patterns of sensory processing and social participation, highlighting the potential relevance of sensory processing to comprehensive assessment and to understanding everyday social participation difficulties.

## Data Availability

The raw data supporting the conclusions of this article will be made available by the authors, without undue reservation.

## References

[1] American Psychiatric Association Force DSMT . Diagnostic and Statistical Manual of Mental Disorders: DSM-5. 5th ed. Arlington, VA: American Psychiatric Publishing, Inc (2013).

[2] Faraone SV Banaschewski T Coghill D Zheng Y Biederman J Bellgrove MA . The world federation of ADHD international consensus statement: 208 evidence-based conclusions about the disorder. Neurosci Biobehav Rev. (2021) 128:789–818. doi: 10.1016/j.neubiorev.2021.01.022 33549739 PMC8328933

[3] Song P Zha M Yang Q Zhang Y Li X Rudan I . The prevalence of adult attention-deficit hyperactivity disorder: A global systematic review and meta-analysis. J Global Health. (2021) 11:4009. doi: 10.7189/jogh.11.04009 33692893 PMC7916320

[4] Aljadani AH Alshammari TS Sadaqir RI Alrashede NOE Aldajani BM Almehmadi SA . Prevalence and risk factors of attention deficit-hyperactivity disorder in the Saudi population: a systematic review and meta-analysis. Saudi J Med Med Sci. (2023) 11:126–34. doi: 10.4103/sjmms.sjmms_528_22 37252016 PMC10211419

[5] Alghamdi WA Alzaben FN Alhashemi HH Shaaban SS Fairaq KM Alsuliamani AS . Prevalence and correlates of attention deficit hyperactivity disorder among college students in Jeddah, Saudi Arabia. Saudi J Med Med Sci. (2022) 10:131–8. doi: 10.4103/sjmms.sjmms_654_21 35602395 PMC9121697

[6] Alharbi N Alotaibi K Althaqel G Alasmari N Alahmari A Alasmari O . Adult Attention Deficit Hyperactivity Disorder (ADHD) among residents of Saudi Arabia: a cross-sectional study. Eur Rev Med Pharmacol Sci. (2023) 27:10935–43. doi: 10.26355/eurrev_202311_34461 38039023

[7] Alsafar FA Alsaad AJ Albukhaytan WA . Prevalence of adult attention deficit hyperactivity disorder (ADHD) among medical students in the Eastern Province of Saudi Arabia. Saudi Med J. (2024) 45:397. doi: 10.15537/smj.2024.45.4.20230841 38657995 PMC11147571

[8] Choi W-S Woo YS Wang S-M Lim HK Bahk W-M . The prevalence of psychiatric comorbidities in adult ADHD compared with non-ADHD populations: A systematic literature review. PLoS One. (2022) 17:e0277175. doi: 10.1371/journal.pone.0277175 36331985 PMC9635752

[9] Kessler RC Adler L Barkley R Biederman J Conners CK Demler O . The prevalence and correlates of adult ADHD in the United States: results from the National Comorbidity Survey Replication. Am J Psychiatry. (2006) 163:716–23. doi: 10.1176/ajp.2006.163.4.716 16585449 PMC2859678

[10] Yavuzlar Civan H Berkol TD . ADHD comorbidity in women with depression and anxiety: prevalence, clinical features and hyperfocus dynamics. Int J Psychiatry Med. (2026) 61:428–44. doi: 10.1177/00912174251376111 40934870

[11] Fu X Wu W Wu Y Liu X Liang W Wu R . Adult ADHD and comorbid anxiety and depressive disorders: a review of etiology and treatment. Front Psychiatry. (2025) 16:1597559. doi: 10.3389/fpsyt.2025.1597559 40547117 PMC12179154

[12] Rice F Riglin L Thapar AK Heron J Anney R O’Donovan MC . Characterizing developmental trajectories and the role of neuropsychiatric genetic risk variants in early-onset depression. JAMA Psychiatry. (2019) 76:306–13. doi: 10.1001/jamapsychiatry.2018.3338 30326013 PMC6439821

[13] Lee L-T Tsai HC Chi MH Chang WH Chen KC Lee IH . Lower availability of striatal dopamine transporter in generalized anxiety disorder: a preliminary two-ligand SPECT study. Int Clin Psychopharmacol. (2015) 30:175–8. doi: 10.1097/yic.0000000000000067 25647452

[14] Barkley RA Brown TE . Unrecognized attention-deficit/hyperactivity disorder in adults presenting with other psychiatric disorders. CNS Spectrums. (2008) 13:977–84. doi: 10.1017/S1092852900014036 19037178

[15] Adamis D West S Singh J Hanley L Coada I McCarthy G . Functional impairment and quality of life in newly diagnosed adults attending a tertiary ADHD clinic in Ireland. Irish J Med Sci (1971-). (2024) 193:2433–41. doi: 10.1007/s11845-024-03713-6 38755511 PMC11457577

[16] Agarwal R Goldenberg M Perry R IsHak WW . The quality of life of adults with attention deficit hyperactivity disorder: a systematic review. Innov Clin Neurosci. (2012) 9:10. PMC3398685 22808445

[17] Ben-Dor Cohen M Eldar E Maeir A Nahum M . Emotional dysregulation and health related quality of life in young adults with ADHD: a cross sectional study. Health Qual Life Outcomes. (2021) 19:270. doi: 10.1186/s12955-021-01904-8 34930314 PMC8691086

[18] Newark PE Elsässer M Stieglitz R-D . Self-esteem, self-efficacy, and resources in adults with ADHD. J Attention Disord. (2016) 20:279–90. doi: 10.1177/1087054712459561 23074301

[19] Arnold LE Hodgkins P Kahle J Madhoo M Kewley G . Long-term outcomes of ADHD: academic achievement and performance. J Attention Disord. (2020) 24:73–85. doi: 10.1177/1087054714566076 25583985

[20] Erskine HE Norman RE Ferrari AJ Chan GC Copeland WE Whiteford HA . Long-term outcomes of attention-deficit/hyperactivity disorder and conduct disorder: a systematic review and meta-analysis. J Am Acad Child Adolesc Psychiatry. (2016) 55:841–50. doi: 10.1016/j.jaac.2016.06.016 27663939

[21] Boop C Cahill SM Davis C Dorsey J Gibbs V Herr B . Occupational therapy practice framework: Domain and process fourth edition. AJOT: Am J Occup Ther. (2020) 74:1–85. doi: 10.5014/ajot.2020.74S2001 34780625

[22] Even-Simkin E . Assessment of pragmatic skills in adults with ADHD. Lang Health. (2024) 2:66–78. doi: 10.1016/j.laheal.2024.02.00242574925

[23] Kessler PB Ikuta T . Pragmatic deficits in attention deficit/hyperactivity disorder: systematic review and meta-analysis. J Attention Disord. (2023) 27:812–21. doi: 10.1177/10870547231161534 36975307

[24] Köder F Rummelhoff C Garraffa M . Comparing pragmatic abilities across multiple languages in adults with ADHD: Insights from a self-report questionnaire. Clin Ling Phonetics. (2025) 39:827–42. doi: 10.1080/02699206.2024.2374909 39016081

[25] Ben-Avi N Almagor M Engel-Yeger B . Sensory processing difficulties and interpersonal relationships in adults: an exploratory study. Psychology. (2012) 3:70–7. doi: 10.4236/psych.2012.31012

[26] Costa-Lopez B Ferrer-Cascales R Ruiz-Robledillo N Albaladejo-Blazquez N Baryła-Matejczuk M . Relationship between sensory processing and quality of life: A systematic review. J Clin Med. (2021) 10:3961. doi: 10.3390/jcm10173961 34501408 PMC8432132

[27] Dunn W Westman K . The sensory profile: the performance of a national sample of children without disabilities. Am J Occup Ther. (1997) 51:25–34. doi: 10.5014/ajot.51.1.25 8978860

[28] Adra N Cao A Makris N Valera EM . Sensory modulation disorder and its neural circuitry in adults with ADHD: a pilot study. Brain Imaging Behav. (2021) 15:930–40. doi: 10.1007/s11682-020-00302-w 32770315 PMC10655817

[29] Bijlenga D Tjon-Ka-Jie J Schuijers F Kooij J . Atypical sensory profiles as core features of adult ADHD, irrespective of autistic symptoms. Eur Psychiatry. (2017) 43:51–7. doi: 10.1016/j.eurpsy.2017.02.481 28371743

[30] Jurek L Duchier A Gauld C Hénault L Giroudon C Fourneret P . Sensory processing in individuals with attention-deficit/hyperactivity disorder compared with control populations: A systematic review and meta-analysis. J Am Acad Child Adolesc Psychiatry. (2025) 64(10):1132–47. doi: 10.1016/j.jaac.2025.02.019 40250555

[31] Kamath M Dahm C Tucker J Huang-Pollock C Etter N Neely K . Sensory profiles in adults with and without ADHD. Res Dev Disabil. (2020) 104:103696. doi: 10.1016/j.ridd.2020.103696 32526674 PMC7786815

[32] Dunn W . The sensations of everyday life: Empirical, theoretical, and pragmatic considerations. Am J Occup Ther. (2001) 55:608–20. doi: 10.5014/ajot.55.6.608 12959225

[33] Adamou M Asherson P Arif M Buckenham L Cubbin S Dancza K . Recommendations for occupational therapy interventions for adults with ADHD: a consensus statement from the UK adult ADHD network. BMC Psychiatry. (2021) 21:72. doi: 10.1186/s12888-021-03070-z 33541313 PMC7863422

[34] Grinblat N Rosenblum S . Work participation, sensory processing and sleep quality in adults with attention-deficit hyperactive disorder. Work. (2022) 73:1235–44. doi: 10.3233/wor-211129 35694942

[35] Lane SJ Reynolds S . Sensory over-responsivity as an added dimension in ADHD. Front Integr Neurosci. (2019) 13:40. doi: 10.3389/fnint.2019.00040 31555103 PMC6742721

[36] Crasta JE Green OJ Gavin WJ Davies PL . The relationship between attention, sensory processing, and social responsiveness among adults on the autism spectrum. J Autism Dev Disord. (2024) 54:2972–86. doi: 10.1007/s10803-023-06019-1 37270447

[37] Pike C Kritzinger A Pillay B . Social participation in working-age adults with aphasia: an updated systematic review. Top Stroke Rehabil. (2017) 24:627–39. doi: 10.1080/10749357.2017.1366012 28851257

[38] Prieur Chaintré A Couturier Y Nguyen TT Levasseur M . Influence of hearing loss on social participation in older adults: results from a scoping review. Res Aging. (2024) 46:72–90. doi: 10.1177/01640275231174561 37157996 PMC10666503

[39] Brown C Tollefson N Dunn W Cromwell R Filion D . The adult sensory profile: Measuring patterns of sensory processing. Am J Occup Ther. (2001) 55:75–82. doi: 10.5014/ajot.55.1.75 11216370

[40] Almomani FM Brown C Dahab SA Almomani M Nadar M . Cross cultural adaptation of the adolescent/adult sensory profile: establishing linguistic equivalency and psychometric properties of the Arabic version. Disability Rehabil. (2014) 36:765–70. doi: 10.3109/09638288.2013.819386 23889527

[41] Brown T Almiento L Yu M-L Bhopti A . The sensory processing measure–second edition: A critical review and appraisal. Occup Ther Health Care. (2024) 38:842–75. doi: 10.1080/07380577.2023.2280216 37975837

[42] Parham LD Ecker CL Kuhaneck HM Henry DA Glennon TJ . Sensory processing measure, (SPM-2). Torrance, CA: Western Psychological Services. (2021).

[43] Alotaibi HM Alduais A Qasem F Alasmari M . Diagnostic and psychometric properties of the arabic sensory processing measure—Second edition, adult version. J Clin Med. (2025) 14:3283. doi: 10.3390/jcm14103283 40429278 PMC12112390

[44] Kessler RC Adler L Ames M Demler O Faraone S Hiripi E . The World Health Organization Adult ADHD Self-Report Scale (ASRS): a short screening scale for use in the general population. Psychol Med. (2005) 35:245–56. doi: 10.1017/s0033291704002892 15841682

[45] Moussa SS Nashaat M . The Arabic Version of the World Health Organization Adult ADHD Self-Report Scale (Asrs) Symptom Checklist for Use in the General Population. Geneva, Switzerland: World Health Organization (2013).

[46] Adler LA Spencer T Faraone SV Kessler RC Howes MJ Biederman J . Validity of pilot Adult ADHD Self-Report Scale (ASRS) to rate adult ADHD symptoms. Ann Clin Psychiatry. (2006) 18:145–8. doi: 10.1080/10401230600801077 16923651

[47] Alqahtani MM Al Saud NM Alsharef NM AlHadi AN Alsalhi SM Al-Hifthy EH . Standardization of the arabic version of the adult ADHD self-report screening scale for DSM-5 (ASRS-5) among adults in saudi arabia: variability of ADHD screening according to sociodemographic variables. J Attention Disord. (2025) 29:445–57. doi: 10.1177/10870547251313879 39851051 PMC11894827

[48] Assaf M Kheir N Hallit R Malaeb D Jebreen K Sakr F . Psychometric assessment of the arabic translation of the adult ADHD self‐Report scale version 1.1 6‐Question screener. Int J Methods Psychiatr Res. (2026) 35:e70083. doi: 10.1002/mpr.70083 42175808 PMC13240305

[49] Cohen J . Statistical Power Analysis for the Behavioral Sciences. 2nd ed. New York: Routledge (1988).

[50] Panagiotidi M Overton PG Stafford T . The relationship between sensory processing sensitivity and attention deficit hyperactivity disorder traits: A spectrum approach. Psychiatry Res. (2020) 293:113477. doi: 10.1016/j.psychres.2020.113477 33198048

[51] Fabio RA Orsino C Lecciso F Levante A Suriano R . Atypical sensory processing in adolescents with attention deficit hyperactivity disorder: A comparative study. Res Dev Disabil. (2024) 146:104674. doi: 10.1016/j.ridd.2024.104674 38306842

[52] Little LM Dean E Tomchek S Dunn W . Sensory processing patterns in autism, attention deficit hyperactivity disorder, and typical development. Phys Occup Ther In Pediatr. (2018) 38:243–54. doi: 10.1080/01942638.2017.1390809 29240517

[53] Lufi D Tzischinsky O . The relationships between sensory modulation and sleep among adolescents with ADHD. J Atten Disord. (2014) 18:646–53. doi: 10.1177/1087054712457036 22923780

[54] Engelhardt PE Ferreira F Nigg JT . Language production strategies and disfluencies in multi-clause network descriptions: A study of adult attention-deficit/hyperactivity disorder. Neuropsychology. (2011) 25:442. doi: 10.1037/a0022436 21463044 PMC5189676

[55] Ginapp CM Greenberg NR Macdonald-Gagnon G Angarita GA Bold KW Potenza MN . The experiences of adults with ADHD in interpersonal relationships and online communities: A qualitative study. SSM-Qualitative Res Health. (2023) 3:100223. doi: 10.1016/j.ssmqr.2023.100223 37539360 PMC10399076

[56] Michielsen M Comijs HC Aartsen MJ Semeijn EJ Beekman ATF Deeg DJH . The relationships between ADHD and social functioning and participation in older adults in a population-based study. J Attention Disord. (2015) 19:368–79. doi: 10.1177/1087054713515748 24378286

[57] Murray A . Suspected and diagnosed ADHD quality of life differences: An investigative study of New Zealand adults. Discover Psychol. (2025) 5:149. doi: 10.1007/s44202-025-00493-730311153

[58] Andersson H Sutton D Bejerholm U Argentzell E . Experiences of sensory input in daily occupations for people with serious mental illness. Scand J Occup Ther. (2021) 28:446–56. doi: 10.1080/11038128.2020.1778784 32544360

[59] Bagatell N Chan DV Syu Y-C Lamarche EM Klinger LG . Sensory processing and community participation in autistic adults. Front Psychol. (2022) 13:876127. doi: 10.3389/fpsyg.2022.876127 35719479 PMC9201716

[60] Thye MD Bednarz HM Herringshaw AJ Sartin EB Kana RK . The impact of atypical sensory processing on social impairments in autism spectrum disorder. Dev Cognit Neurosci. (2018) 29:151–67. doi: 10.1016/j.dcn.2017.04.010 28545994 PMC6987885

[61] Forsberg K Sutton D Stjernswärd S Brown C Bejerholm U Argentzell E . Sensory processing patterns and their relationships to coping and occupational engagement in mental health service users. Aust Occup Ther J. (2025) 72:e13016. doi: 10.1111/1440-1630.13016 39835693 PMC11748359

[62] Martin J . Why are females less likely to be diagnosed with ADHD in childhood than males? Lancet Psychiatry. (2024) 11:303–10. doi: 10.1016/S2215-0366(24)00010-5 38340761

[63] Staley BS Robinson LR Claussen AH Katz SM Danielson ML Summers AD . Attention-deficit/hyperactivity disorder diagnosis, treatment, and telehealth use in adults—National Center for Health Statistics rapid surveys system, United States, October–November 2023. MMWR Morbidity Mortality Weekly Rep. (2024) 73:890–5. doi: 10.15585/mmwr.mm7340a1 39388378 PMC11466376

[64] Adamou M Jones SL . Adult ADHD diagnostic pathways in the United Kingdom: A structured review and policy case for a national commissioning and quality framework for England. Front Psychiatry. (2026) 17. doi: 10.3389/fpsyt.2026.1860550 42553636 PMC13435771

[65] Alkathiry A Alrashed R Alfadhly R Al-Tannir M . Knowledge, attitude, and performance of primary healthcare physicians toward diagnosis and management of attention deficit hyperactivity disorder at PHC centers in Riyadh second health cluster, Saudi Arabia. J Family Med Prim Care. (2026) 15:1322–30. doi: 10.4103/jfmpc.jfmpc_1754_25 42257142 PMC13241152

[66] Dunlop BW Wu R Helms K . Performance of the adult ADHD self-report scale-v1. 1 in adults with major depressive disorder. Behav Sci. (2018) 8:37. doi: 10.3390/bs8040037 29596328 PMC5946096

[67] Choi BC Pak AW . A catalog of biases in questionnaires. Preventing Chronic Dis. (2004) 2:A13. PMC1323316 15670466

[68] Podsakoff PM MacKenzie SB Lee J-Y Podsakoff NP . Common method biases in behavioral research: A critical review of the literature and recommended remedies. J Appl Psychol. (2003) 88:879. doi: 10.1037/0021-9010.88.5.879 14516251

[69] Alhumaidan NI Alotaibi TA Aloufi KS Althobaiti AA Althobaiti NSA Althobaiti K . Barriers to seeking mental health help in Saudi Arabia: A systematic review. Cureus. (2024) 16:e60363. doi: 10.7759/cureus.60363 38883041 PMC11178508

[70] Skocic D Brown T Yu ML Reed K . Convergent validity of two adult self‐report sensory scales: Comparing the Adolescent/Adult Sensory Profile and the Sensory Processing Measure 2–Adult Form. Aust Occup Ther J. (2024) 71:897–909. doi: 10.1111/1440-1630.12963 38738292 PMC11609396

