# Supplementary material for: Self-reported sensory processing and social participation across adult ADHD symptom groups
Source: Front Psychiatry. 2026 Sep 29;17:1928930. doi: 10.3389/fpsyt.2026.1928930 (PMC13658455; doi:10.3389/fpsyt.2026.1928930)
Supplement: Supplementary file 1 [file Table1.docx]

| **Supplementary Table S1**  *Internal Consistency Estimates for Outcome Measures in the Present Sample* | | | |
| --- | --- | --- | --- |
| **Measure or subscale** | **Items** | **N** | **Cronbach’s α** |
| ASRS Part A | 6 | 209 | .75 |
| AASP Low Registration | 15 | 209 | .88 |
| AASP Sensation Seeking | 15 | 209 | .66 |
| AASP Sensory Sensitivity | 15 | 209 | .79 |
| AASP Sensation Avoiding | 15 | 209 | .80 |
| SPM-2 Social Participation | 10 | 209 | .77 |
| *Note.* Internal consistency was estimated using raw Cronbach’s alpha based on item-level responses. AASP = Adolescent/Adult Sensory Profile; ASRS = Adult ADHD Self-Report Scale; ADHD = attention-deficit/hyperactivity disorder; SPM-2 = Sensory Processing Measure–2. | | | |

| **Supplementary Table S2**  *Anxiety & Depression Cross-classification* | | | | | |
| --- | --- | --- | --- | --- | --- |
| **Study group** | **Both anxiety and depression** | **Anxiety only** | **Depression only** | **Neither** | **Total N** |
| Self-reported ADHD diagnosis | 38 | 19 | 8 | 26 | 91 |
| Elevated ADHD symptoms | 9 | 11 | 1 | 45 | 66 |
| Low ADHD symptoms | 2 | 2 | 2 | 46 | 52 |
| *Note.* Values represent cross-classified frequencies of self-reported anxiety and depression diagnoses within each study group. | | | | | |

| **Supplementary Table S3**  *ADHD Diagnosis and Treatment Characteristics Among Participants with Self-reported ADHD diagnosis* | |  |
| --- | --- | --- |
| **Characteristic** | **N = 91**^1^ | |
| Age at formal ADHD diagnosis | 25.00 (21.00, 31.00); range: 5.00–45.00 | |
| Previously received ADHD medication or intervention | 79 (86.8%) | |
| Stimulant medication for ADHD | 63 (70.0%) | |
| Non-stimulant medication for ADHD | 31 (35.2%) | |
| Behavioral therapy for ADHD | 33 (36.7%) | |
| Cognitive behavioral therapy for ADHD | 35 (38.9%) | |
| *Note.* Participants could select multiple treatment options.1Median (Q1, Q3); range: Min–Max; n (%) | | |

| **Supplementary Table S4**  *ASRS Part A Screening Status* | | |
| --- | --- | --- |
| **Study group** | **ASRS Part A < 4** | **ASRS Part A ≥ 4** |
| Self-reported ADHD diagnosis | 3 (3.3%) | 88 (96.7%) |
| Self-suspected ADHD, no diagnosis | 9 (14.5%) | 53 (85.5%) |
| No self-suspected ADHD, no diagnosis | 43 (76.8%) | 13 (23.2%) |
| *Note.* Values are presented as n (%); percentages were calculated within each row. N = 209. ASRS = Adult ADHD Self-Report Scale; ADHD = attention-deficit/hyperactivity disorder. ASRS Part A ≥ 4 indicates elevated ADHD symptoms based on the ASRS v1.1 screener scoring threshold. | | |

| **Supplementary Table S5**  *Sensitivity Analysis - ANCOVA* | | | | |
| --- | --- | --- | --- | --- |
| **Outcome** | **F (df1, df2)** | **Raw p** | **Partial η²** | **BH-adjusted p** |
| Low registration | 44.36 (2, 204) | <.001 | 0.403 | <.001 |
| Sensation seeking | 5.20 (2, 204) | 0.006 | 0.050 | 0.006 |
| Sensory sensitivity | 25.23 (2, 204) | <.001 | 0.285 | <.001 |
| Sensation avoiding | 15.46 (2, 204) | <.001 | 0.224 | <.001 |
| Social participation | 18.89 (2, 204) | <.001 | 0.236 | — |
| *Note*. This table presents the omnibus study-group effect from adjusted group-comparison models with anxiety and depression entered as separate covariates (sensitivity analysis). Compare with Table 2 in the main manuscript, which reports results using the combined anxiety/depression covariate. BH-adjusted p values were calculated across the four AASP quadrant outcomes. | | | | |

| **Supplementary Table S6**  *Sensitivity Analysis - Hierarchical Regression* | | | | | | | | |
| --- | --- | --- | --- | --- | --- | --- | --- | --- |
| **Predictor** | **Model 1: Covariates Only** | | | | **Model 2: Combined Sensory Model** | | | |
|  | **B** | **SE** | **p** |  | **B** | **SE** | **p** |  |
| Intercept | 21.72 | 0.86 | <.001 |  | 14.37 | 2.84 | <.001 |  |
| Self-reported ADHD diagnosis | 7.35 | 1.23 | <.001 |  | 2.04 | 1.20 | .090 |  |
| Elevated ADHD symptoms | 5.44 | 1.17 | <.001 |  | 1.13 | 1.08 | .299 |  |
| Anxiety | 1.90 | 1.11 | .090 |  | 0.07 | 0.93 | .940 |  |
| Depression | 0.29 | 1.18 | .808 |  | 0.02 | 0.96 | .980 |  |
| q1: Low registration | — | — | — |  | 0.28 | 0.05 | <.001 |  |
| q2: Sensation seeking | — | — | — |  | -0.19 | 0.05 | <.001 |  |
| q3: Sensory sensitivity | — | — | — |  | 0.06 | 0.06 | .342 |  |
| q4: Sensation avoiding | — | — | — |  | 0.12 | 0.06 | .037 |  |
| **Model fit summary** | | | | | | | | |
| R² | .248 |  |  |  | .514 |  |  |  |
| Adjusted R² | .233 |  |  |  | .495 |  |  |  |
| ΔR² | — |  |  |  | .266 |  |  |  |
| ΔF | — |  |  | F(4, 200) = 27.38, *p* < .001 | | | |  |
| Note. This table presents the hierarchical regression predicting Social Participation with anxiety and depression entered as separate covariates (sensitivity analysis). Compare with Table 3 in the main manuscript, which reports results using the combined anxiety/depression covariate. | | | | | | |  |  |
